# Association Between Polymorphisms in Genes Encoding PD-1/PD-L1 Molecules and Clinicopathological Features in Clear Cell Renal Cell Carcinoma

**DOI:** 10.3390/ijms27083435

**Published:** 2026-04-11

**Authors:** Magdalena Onyszczuk, Nikola Szweda-Gandor, Magdalena Rynkiewicz, Bogna Drozdzowska

**Affiliations:** 1 Department of Pathomorphology, Faculty of Medical Sciences in Zabrze, Medical University of Silesia, 40-055 Katowice, Poland; mrynkiewicz@sum.edu.pl (M.R.); bognadr@poczta.onet.pl (B.D.); 2 Department and Clinic of Internal Medicine, Diabetology and Nephrology, Medical University of Silesia, 41-800 Zabrze, Poland; nikola.szweda@gmail.com

**Keywords:** *CD274*, clear cell renal cell carcinoma, immune checkpoint molecules, genetic association, PD-1, *PDCD1*, PDL-1, single nucleotide polymorphism

## Abstract

The PD-1/PD-L1 axis is crucial for immune regulation and homeostasis, but cancer cells can exploit this pathway to evade immune surveillance. PD-1, a key immune checkpoint receptor, interacts with its ligands PD-L1 and PD-L2 to modulate immune responses within the tumor microenvironment. We hypothesized that single nucleotide polymorphisms (SNPs) in the *PDCD1* and *CD274* genes, encoding PD-1 and PD-L1, are associated with clinicopathological features, PD-L1 immunohistochemical expression, and clinical outcomes in clear cell renal cell carcinoma (ccRCC). We analyzed four SNPs using TaqMan allelic discrimination assays in 238 ccRCC cases: rs11568821 and rs7603052 (*PDCD1*), and rs4143815 and rs17718883 (*CD274*). The rs7603052 polymorphism in *PDCD1* and rs17718883 in *CD274* were significantly associated (*p* = 0.033 and *p* = 0.043 respectively) with PD-L1 expression in tumor-infiltrating immune cells (TIICs). Specifically, the C allele of rs7603052 and the CC genotype of rs17718883 correlated with PD-L1 positivity in TIICs. Additionally, the C allele of rs4143815 in CD274 was associated with PD-L1 positivity in tumor cells (*p* = 0.039). Notably, rs17718883 in *CD274* was associated with ccRCC patient prognosis: carriers of the T allele, particularly those with the CT genotype, demonstrated improved overall survival compared to CC genotype carriers (*p* < 0.001). These findings suggest that *PDCD1* and *CD274* polymorphisms may serve as potential predictive and prognostic biomarkers in ccRCC.

## 1. Introduction

A key characteristic of cancer, as described by Hanahan and Weinberg [1], is the ability of tumor cells to evade immune surveillance. In order to avoid immune detection, cancer cells utilize multiple strategies, one of the most important being the development of an immunosuppressive tumor microenvironment. This is largely mediated by the expression of immune checkpoint molecules, which have inhibitory effects on immune function [2]. Among these, programmed death protein 1 (PD-1) and its ligands, programmed death ligands (PD-Ls), are considered central regulators of immune responses. PD-1 is a transmembrane receptor belonging to the CD28/CTLA-4 family and is widely expressed on activated immune cells, including T cells, B cells, and monocytes. It acts as a negative regulator of the immune system through interactions with its ligands, PD-L1 (B7-H1; CD274) and PD-L2 (B7-DC; CD273). When PD-1 binds to either ligand, inhibitory signals are transmitted that reduce T-cell activity, promote immune tolerance, and help maintain immune homeostasis [3]. Importantly, these ligands can also be expressed by tumor and stromal cells. Within the tumor microenvironment, activation of the PD-1/PD-L1 pathway suppresses T-cell function, weakening the immune response against cancer and enabling tumor cells to avoid immune surveillance [4,5]. Engagement of PD-1 on tumor-infiltrating lymphocytes with PD-L1 on tumor or other immune cells triggers signaling cascades that suppress immune activity. Blocking this interaction can reverse T-cell inhibition, restoring their ability to recognize and eliminate cancer cells. Therapies targeting this pathway, known as immune checkpoint inhibitors (ICIs), have therefore emerged as an effective approach to cancer treatment. By preventing tumors from exploiting checkpoint mechanisms, these agents enhance the immune system’s capacity to identify and attack malignant cells [6,7]. Over the past decade, ICIs targeting both PD-1 and PD-L1 have been incorporated into clinical practice and have shown significant therapeutic benefit across multiple cancer types, often outperforming conventional chemotherapy. However, not all patients respond to this therapy, and some experience considerable adverse effects, making appropriate patient selection for immunotherapy a critical challenge [6]. Renal cell carcinoma (RCC) is one of the malignancies traditionally regarded as “immunotherapy-responsive” [8]. This study focuses on clear cell RCC (ccRCC), the most prevalent RCC subtype, accounting for approximately 70–80% of RCC cases.

Current evidence suggests that both tumor development and clinical progression may be influenced by the expression levels of immune checkpoint molecules, including members of the PD-1/PD-L1 axis. It is well-established that the mRNA and protein expression levels can be affected by genetic variations that alter epigenetic regulation, transcription factor binding sites, and the formation of protein isoforms [9]. As a result, genetic polymorphisms impacting PD-1 and PD-L1 expression or function have been investigated in various types of cancers. Nevertheless, there is limited data specifically concerning RCC. In light of this, the present study was designed to investigate potential associations between single nucleotide polymorphisms (SNPs) in the genes encoding PD-1 and PD-L1 molecules and their correlation with clinicopathological characteristics, PD-L1 immunohistochemical expression, and clinical outcomes in ccRCC cases. Four SNPs were selected for analysis: two within the *PDCD1* gene (encoding PD-1 molecule) and two within the *CD274* gene (encoding PD-L1 molecule).

## 2. Results

### 2.1. PDCD1 and CD274 Polymorphisms

In the present study, we investigated four polymorphisms—two located in the *PDCD1* gene (encoding the PD-1 molecule): rs11568821 and rs7603052, and two in the *CD274* gene (encoding the PD-L1 molecule): rs4143815 and rs17718883. Table 1 and Supplemental Figures S1 and S2 present the allele frequencies and genotype distributions of the examined SNPs in ccRCC cases. For all SNPs, the genotype distributions were consistent with the Hardy–Weinberg equilibrium.

### 2.2. Association of PDCD1 and CD274 Genes Polymorphisms with Clinicopathological Features

In this study, we assessed the effects of *PDCD1* and *CD274* polymorphisms on patients’ sex and age, tumor size, presence of angioinvasion, WHO/ISUP grade, and AJCC TNM pathological stage of the primary tumor (pT).

No significant associations were observed between the investigated SNPs and the above-mentioned clinicopathological features (Supplemental Tables S1–S6).

### 2.3. Association of PDCD1 and CD274 Genes Polymorphisms with PD-L1 Immunohistochemical Expression in Tumor-Infiltrating Immune Cells (TIICs)

We observed that two of the four examined SNPs appeared to be associated with PD-L1 immunohistochemical expression in TIICs. A significant difference in genotype distribution was found for rs7603052 in *PDCD1* and rs17718883 in *CD274*. Specifically, C allele carriers (TC and CC genotypes) of rs7603052 were more frequent among individuals with PD-L1 positivity in TIICs compared to TT homozygotes, suggesting a positive association between the C allele of rs7603052 and PD-L1 expression in TIICs (*p* = 0.033). Similarly, rs17718883 CC homozygotes were more frequent in the group with PD-L1 positivity in TIICs compared to T allele carriers (CT and TT genotypes), indicating a positive association between the rs17718883 CC genotype and PD-L1 expression in TIICs (*p* = 0.043). However, it should be noted that, although these associations reached nominal statistical significance (*p* < 0.05), they did not remain significant after correction for multiple testing after False Discovery Rate (FDR) adjustment.

No evidence of an association between the remaining SNPs and PD-L1 immunohistochemical expression in TIICs was observed.

The associations between PDCD1 and CD274 gene polymorphisms and PD-L1 immunohistochemical expression in TIICs are presented in Table 2 and Supplemental Figure S3.

### 2.4. Association of PDCD1 and CD274 Genes Polymorphisms with PD-L1 Immunohistochemical Expression in ccRCC Tumor Cells

In this analysis, one of the four examined SNPs appeared to be associated with PD-L1 immunohistochemical expression in ccRCC tumor cells. A significant difference in genotype distribution was observed for rs4143815 in *CD274*. C allele carriers (GC and CC genotypes) of rs4143815 were more frequent in the group with PD-L1-positive tumor cells compared to GG homozygotes, suggesting a positive association between the C allele of rs4143815 and PD-L1 expression in tumor cells (*p* = 0.039). However, it should be noted that, although this association reached nominal statistical significance (*p* < 0.05), it did not remain significant after correction for multiple testing after FDR adjustment.

No evidence of an association between the other SNPs and PD-L1 immunohistochemical expression in tumor cells was observed.

The associations between *PDCD1* and *CD274* gene polymorphisms and PD-L1 immunohistochemical expression in tumor cells are presented in Table 3 and Supplemental Figure S4.

### 2.5. PDCD1 and CD274 Polymorphisms and Overall Survival (OS) of ccRCC Patients

**Figure 1:**
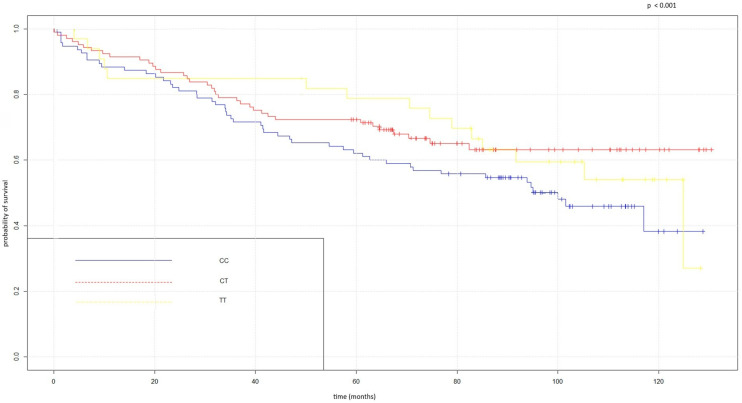
Kaplan–Meier overall survival curves for rs17718883 in the *CD274* gene.

We observed that one of the four examined SNPs—rs17718883 in the *CD274* gene—was associated with OS in ccRCC patients. The analysis revealed that T allele carriers (particularly those with the CT genotype) of rs17718883 had more favorable OS compared to patients with the CC genotype (Figure 1). No association between the remaining SNPs and OS was observed.

Table 4 and Supplemental Figure S5 present the associations between the genotypes of the investigated SNPs and OS in ccRCC patients.

## 3. Discussion

Carcinogenesis is a complex, multistage, and multifactorial process resulting from interactions between environmental exposures and genetic predisposition [10]. A growing body of evidence highlights the pivotal role of the immune system in identifying and eliminating malignant cells. T lymphocytes are the most important cells to the antitumor immune response, contributing to both cancer initiation and progression [11]. Their activation and expansion are tightly regulated by a balance of co-stimulatory and co-inhibitory signals mediated by members of the CD28/B7 family [12]. In this context, genes encoding PD-1 and its ligands have drawn significant attention due to their key role in preserving immune tolerance [13]. SNPs are the most common type of genetic variation linked to cancer susceptibility and progression. They serve as valuable biomarkers for identifying genes involved in complex diseases, including malignancies [14]. Consequently, SNPs in genes related to immune regulation—particularly those influencing T-cell activity—may therefore contribute to cancer development and progression [8]. Numerous studies have shown that polymorphisms within genes of the PD-1/PD-L1 pathway, especially in regulatory regions such as the promoter and 3′-untranslated region (3′-UTR), can significantly affect PD-1 and PD-L1 expression [15,16]. These variants may alter post-transcriptional regulation, including interactions with microRNAs (miRNAs), thereby influencing cancer risk, prognosis, and response to therapies such as chemotherapy or immune checkpoint blockade [17,18,19]. Based on these observations, our study focused on selected polymorphisms in PD-1 and PD-L1 genes that have previously been investigated across different cancer types.

The rs17718883 polymorphism is a relatively common SNP in the *CD274* gene that leads to a proline-to-arginine substitution at position 146 (Pro146Arg; P146R), representing a missense mutation. This polymorphism is present in approximately 17–46% of healthy individuals. Structural analyses using molecular dynamics simulations suggest that the P146R substitution disrupts hydrogen bonding networks and increases the distance between protomers at the PD-L1 dimer interface, thereby destabilizing PD-L1 formation. These structural alterations may interfere with PD-1/PD-L1 binding, potentially affecting disease progression in gastric cancer and reducing the effectiveness of PD-1/PD-L1–targeted therapies [20]. This example illustrates how genetic variation can substantially influence biological mechanisms relevant to cancer outcomes and treatment response. However, these findings are largely based on in silico models and have not yet been directly confirmed in renal cancer models. An increasing number of studies indicate that rs17718883 is associated with lower cancer risk and more favorable prognosis in several malignancies, including gastric cancer, hepatocellular carcinoma, hematopoietic and lymphoid neoplasms, and certain dermatological conditions [20,21,22,23,24]. These observations suggest that this polymorphism may impair PD-L1 function, thereby influencing the course of diseases in which PD-L1 plays a role [20]. Our findings are in line with previous reports. In our cohort, the *CD274* rs17718883 polymorphism showed a significant association with OS—individuals carrying the T allele, particularly those with the CT genotype, had longer OS compared to CC homozygotes. Previous work by Li et al. [20] reported no differences in PD-L1 expression between gastric cancer carrying the P146R variant and those with the wild-type PD-L1. Consistent with this, we did not observe an association between rs17718883 and PD-L1 immunohistochemical expression in tumor cells of ccRCC. However, we find a relationship between this polymorphism and PD-L1 expression in TIICs. These results suggest that rs17718883 may influence cancer outcomes not by altering PD-L1 expression levels directly, but rather by modifying its functional interaction with PD-1 on T cells. Furthermore, Li et al. [20] identified rs17718883 as a negative predictor of response to PD-1/PD-L1 blockade in gastric cancer. This raises the possibility that this SNP could affect responsiveness to ICIs. Nevertheless, its potential predictive value should be confirmed in independent studies with sufficiently large and well-characterized patient cohorts.

The *PDCD1* rs11568821 polymorphism (also known as 7146 G>A or PD-1.3) is a SNP at nucleotide 7146 within intron 4 of the *PDCD1* gene and involves a guanine-to-adenine substitution. This region has been described as an enhancer-like element containing binding sites for several transcription factors. Evidence suggests that this SNP region disrupts the binding site for runt-related transcription factor 1 (RUNX1), potentially altering *PDCD1* transcription and PD-1 signaling efficiency [25,26]. Several studies have investigated the association between rs11568821 and various malignancies, including thyroid cancer, colorectal cancer, hepatocellular carcinoma, chronic lymphocytic leukemia, non-small cell lung cancer, and melanoma [27,28,29,30,31,32,33,34]. To date, only one study—conducted by Wagner et al. [9]—has evaluated this polymorphism in the context of renal cancer. The authors did not observe any association between rs11568821 and the risk of ccRCC development or OS. Our findings corroborate these observations, as we likewise found no association between rs11568821 and prognosis in patients with ccRCC.

The rs7603052 polymorphism is situated within an intronic region of the *PDCD1* gene, and its clinical significance has not been well established. Evidence regarding this variant is limited. For instance, a comprehensive meta-analysis conducted by Yang et al. [35], which included 50 studies investigating PD-1/PD-L1 polymorphisms and cancer susceptibility, did not evaluate rs7603052. To date, only one study has explored the role of rs7603052 in renal cancer. This analysis focused on whether polymorphisms in immune checkpoint–related genes were associated with clinical outcomes in patients with metastatic ccRCC receiving sunitinib as first-line tyrosine kinase inhibitor (TKI) therapy [36]. The results showed no correlation between rs7603052 and OS in this patient group, indicating that this SNP is unlikely to have prognostic or predictive value in renal cancer.

The rs4143815 SNP is located in the 3′-UTR of the *CD274* gene, which encodes the PD-L1 protein. MiRNAs are endogenous, non-coding RNA molecules, typically 19–24 nucleotides long, that regulate gene expression post-transcriptionally by binding to complementary sequences within the 3′-UTR of target mRNAs. Several miRNAs—such as miR-570, miR-7-1, miR-495, and miR-298—have been predicted to interact with the rs4143815 3′-UTR region [37,38,39]. A guanine-to-cytosine substitution within the 3′-UTR of *CD274* mRNA has been reported to increase PD-L1 expression by interfering with miR-570 binding and decreasing mRNA degradation [40,41]. Additionally, the G allele in this position is thought to form a larger stem–loop structure compared to the C allele, which may influence the interaction between the PD-L1 3′-UTR and miR-570, ultimately leading to reduced PD-L1 expression [38]. Wang et al. [41] showed that, in gastric cancer, the C allele may weaken miR-570 binding, thereby promoting higher PD-L1 protein levels. Our findings are consistent with these observations. We found that individuals carrying the C allele (GC and CC genotypes) of rs4143815 were more frequently represented among cases with PD-L1–positive tumor cells compared with GG homozygotes. This suggests that the C allele of rs4143815 is associated with increased PD-L1 expression in tumor cells of ccRCC. Importantly, the presence of the C allele—particularly the CC genotype—may enhance PD-L1 expression and strengthen its interaction with the PD-1 receptor, thereby contributing to T-cell exhaustion. This state is characterized by increased apoptosis, reduced proliferation, and impaired effector function of T cells [40,42]. In another study, patients with non-small-cell lung cancer carrying the GG genotype of rs4143815 did not respond to nivolumab therapy [19]. Taken together, these findings indicate that rs4143815, through its effect on PD-L1 expression, may have potential as a predictive marker for response to immunotherapy. This is important because, to date, there is not a single predictive biomarker approved for selecting patients with RCC who are likely to benefit from ICIs targeting PD-L1 [43]. PD-L1 expression in tumor tissue, assessed using immunohistochemical staining, is an established biomarker for predicting the therapeutic efficacy of ICIs in multiple cancer types and is incorporated into treatment decisions for, among others, urothelial, breast, non-small cell lung, and head and neck cancers [6,44,45]. However, its role in RCC is not clearly defined. ICI therapy has improved clinical outcomes in patients with advanced RCC in clinical trials, and PD-L1 immunohistochemical analysis was performed in these studies using four FDA-approved PD-L1 IHC assays: PD-L1 IHC 22C3 pharmDx (Agilent Technologies, Inc.), PD-L1 IHC 28-8 pharmDx (Agilent Technologies, Inc.), VENTANA PD-L1 (SP142) Assay (Roche Diagnostics K.K.), and VENTANA PD-L1 (SP263) Assay (Roche Diagnostics K.K.) [46]. However, it should be noted that ICIs provide clinical benefit in RCC regardless of PD-L1 expression, as assessed by any antibody, which limits its utility as a biomarker. Overall, PD-L1 expression is not routinely used as a biomarker in RCC [44]. Since none of the PD-L1 antibody clones have been approved as uniquely relevant for predicting the therapeutic efficacy of ICIs in kidney cancer, we performed our immunohistochemical analysis of PD-L1 using a less common anti–PD-L1 monoclonal antibody, clone ZR3, produced by Zeta Corporation, Arcadia, CA, USA. Recent studies have explored the potential association between rs4143815 and susceptibility to various cancers; however, the results remain inconsistent [17,35,47,48,49,50,51]. Some reports suggest that rs4143815 C>G increases cancer risk, whereas others have found no significant association. A meta-analysis conducted by Zou et al. [17], which included 3711 cases and 3704 controls, indicated that rs4143815 C>G was associated with increased overall cancer susceptibility and may elevate the risk of gastric and bladder cancers. Additionally, individuals with the GG genotype might have a higher risk of hepatocellular carcinoma. These findings suggest that rs4143815 could contribute to cancer pathogenesis and potentially serve as a biomarker of cancer risk. Yang et al. [35] performed another meta-analysis of 12 studies comprising 4008 cases and 4147 controls and found no statistically significant association between rs4143815 and overall cancer risk across all genetic models. However, subgroup analyses indicated that this variant was associated with a decreased risk of gastric cancer, hepatocellular carcinoma, and ovarian cancer, but an increased risk of breast cancer. The discrepancies among studies may be explained by differences in study design, limited sample sizes, ethnic heterogeneity, and potential selection bias. To date, the only study investigating rs4143815 in the context of RCC was conducted by Wagner et al. [9]. They demonstrated that the C C G A haplotype (rs822335 rs10815225 rs4143815 rs4742098) was more frequent among ccRCC patients than controls and was associated with an increased risk of developing ccRCC.

Summarizing, the English-language literature provides limited data on polymorphisms in genes encoding the PD-1/PD-L1 axis in kidney cancer. The largest study assessing PD-1/PD-L1 SNPs in relation to RCC susceptibility and prognosis was conducted by Wagner et al. [9]. In that study, nine polymorphisms—five in *PDCD1* and four in *CD274*—were genotyped in 237 RCC patients, including 208 with ccRCC. The authors identified a significant SNP–SNP interaction between rs10815225 in *CD274* and rs7421861 in *PDCD1*, which was associated with an increased risk of ccRCC. These two variants were therefore proposed as potential genetic risk factors for ccRCC. No significant associations were observed for the remaining polymorphisms analyzed, including rs11568821 and rs4143815, which were also examined in our study. Importantly, that research did not explore the relationship between PD-1/PD-L1 polymorphisms and clinicopathological features and PD-L1 immunohistochemical expression in ccRCC patients.

To the best of our knowledge, the present study is the first to evaluate the association between selected polymorphisms in genes encoding the PD-1/PD-L1 axis and clinicopathological features, immunohistochemical PD-L1 expression, and prognosis in patients with ccRCC. Taken together, our findings suggest that genetic variation in the PD-1/PD-L1 axis may contribute to interindividual differences in tumor immune escape and clinical heterogeneity in ccRCC. Nevertheless, several limitations should be acknowledged. First, the study did not include a control group or detailed clinical parameters. Moreover, although some associations reached nominal statistical significance (*p* < 0.05), they did not remain significant after correction for multiple testing, indicating that these findings may be exploratory, should be interpreted as hypothesis-generating, and require validation in larger cohorts. Future prospective clinical studies should validate these signals in independent, adequately powered cohorts and explore functional mechanisms, including expression quantitative trait locus (eQTL) effects, transcript- and protein-level regulation of PD-1/PD-L1, and interactions with the tumor immune microenvironment. Importantly, integrating germline genotypes with detailed treatment data (including response to personalized therapy) and clinically relevant endpoints such as progression-free survival and disease-free survival may help clarify whether these variants have predictive value for therapy response. Moreover, the proposed functional mechanism linking rs4143815 in the 3′-UTR of *CD274* to altered miRNA binding and PD-L1 expression was based on the existing literature and remains hypothetical in our study, as we did not perform any functional validation experiments, such as luciferase reporter assays, quantitative PCR for PD-L1 mRNA, or genotype-stratified correlation analyses with protein expression to support this mechanism. To prove this mechanism further functional studies are needed to confirm whether rs4143815 directly affects miRNA binding and PD-L1 expression. Despite these limitations, our study may serve as a foundation for further discussion and enhance understanding of the clinical implications of genetic alterations in PD-L1- and PD-1–encoding genes in ccRCC cases.

## 4. Materials and Methods

### 4.1. General Patients Characteristics

A total of 238 ccRCC patients (83 females and 155 males) diagnosed in the Department of Pathomorphology at the Medical University of Silesia, Katowice, Poland were included in the present study. Cases with other histological RCC subtypes were excluded. All tumor specimens were obtained during partial or radical nephrectomy performed for sporadic RCC between December 2014 and October 2020. The tissue specimens were formalin-fixed and paraffin-embedded using routine pathological tissue-processing techniques. Detailed clinicopathological characteristics of the patients are presented in Table 5.

The study cohort was followed for all-cause mortality until September 2025. Follow-up data included the date of surgery, survival status, date of last follow-up and/or date of death. The mean duration of follow-up was 71 months (SD = 37), with a median of 75 months.

The Institutional Review Board of the Medical University of Silesia determined that this study did not require approval from the bioethics committee (BNW/NWN/0052/KB/109/23). Anonymized and de-identified data were used for the analyses. The authors did not have access to information that could identify individual participants during or after data collection.

### 4.2. SNP Selection

Based on the bioinformatics database http://www.ncbi.nlm.nih.gov/snp/ (accessed on 26 April 2025) four SNP loci were selected—two located in *PDCD1* gene (encoding the PD-1 molecule): rs11568821 and rs7603052, and two in *CD274* gene (encoding the PD-L1 molecule): rs4143815 and rs17718883. Genotyping was performed using predesigned TaqMan™ SNP Genotyping Assays (Thermo Fisher Scientific, Waltham, MA, USA). The assay IDs were as follows: PDCD1 rs7603052—C_56214_10; PDCD1 rs11568821—C__57931290_10; CD274 rs4143815—C__31941235_10; CD274 rs17718883—predesigned TaqMan SNP Genotyping Assay (assay ID provided in the manufacturer’s Assay Information File). Assay-specific primer/probe sequences for predesigned TaqMan assays are proprietary; the manufacturer provides the context sequence and allele–dye assignment in the Assay Information File.

### 4.3. DNA Extraction and Genotyping

MagCore Genomic DNA FFPE One-Step kit of the MagCore isolation system (FFPE One-Step kit of the MagCore RBC Bioscience Corp, New Taipei City, Taiwan) was used in order to perform necessary genetic tests. The extracted DNA was subsequently used for allelic discrimination of the four selected polymorphisms. Genotyping was performed using the Roche LightCycler^®^ 96 System (Roche, Basel, Switzerland) according to the manufacturer’s protocol. Real-time fluorescent quantitative polymerase chain reaction (PCR) was conducted using the appropriate TaqMan genotyping method (TaqMan Thermo Fisher Scientific Waltham, MA, USA) for allelic discrimination. One blank control (NTC; molecular-grade water) was included in each 96-well plate as a quality control measure. Genotypes were automatically assigned using allelic discrimination and verified using quality control metrics; NTC was included on each plate.

### 4.4. PD-L1 Immunohistochemical Staining and Interpretation

The immunohistochemical staining was performed with the ZR3 clone (Zeta Corporation, Arcadia, CA, USA), using the same methodology described in the authors’ previous study [52]. PD-L1 expression in cancer cells and TIICs was interpreted separately. PD-L1 positivity in cancer cells was defined as the presence of a linear membranous immunostaining pattern in ≥1% of viable tumor cells, regardless of staining intensity. PD-L1 positivity in TIICs was defined as membranous or cytoplasmic staining observed in ≥1% of immune cells (macrophages, dendritic cells, and lymphocytes) at any intensity.

### 4.5. Statistical Analysis

Statistical analysis was performed using Spotfire Statistica^®^ 14.1.04 (TIBCO Software Inc., Palo Alto, CA, USA) and Microsoft Excel (Microsoft Office 2024 Standard; Microsoft Corp., Redmond, WA, USA). Categorical variables were presented as n (%), whereas continuous variables were expressed as mean ± standard deviation (SD) or median (interquartile range, IQR) depending on their distribution. Normality was assessed with the Shapiro–Wilk test. Hardy–Weinberg equilibrium for each SNP was evaluated using the χ^2^ test. Associations between genotypes and categorical variables were assessed using the χ^2^ test (with Yates’ continuity correction when appropriate) or Fisher’s exact test. Differences in continuous variables between genotype groups were evaluated using one-way ANOVA (or Welch ANOVA if variances were unequal); otherwise, the Kruskal–Wallis test was applied. Post hoc comparisons were conducted using Tukey’s HSD (ANOVA) or Dunn’s test (Kruskal–Wallis) with appropriate correction for multiple comparisons. For adjusted analyses, linear regression (for continuous outcomes) and logistic regression (for binary outcomes) models were fitted, with genotypes coded according to additive (0/1/2), dominant, and recessive genetic models and adjusted for predefined covariates (e.g., age and sex). Multiple testing was controlled using the Benjamini–Hochberg FDR. OS was estimated using the Kaplan-Meier method. A log-rank test was used to assess the association between predictor variables and patient survival. A Cox proportional hazards model was used to evaluate the impact of qualitative and continuous variables on survival. All statistical tests were two-sided, and a *p*-value < 0.05 was considered statistically significant.

## 5. Conclusions

In summary, our findings indicate that polymorphisms in genes of the PD-1/PD-L1 axis may influence the clinical course of ccRCC and could serve as potential predictive and prognostic biomarkers in kidney cancer. We observed that rs7603052 in *PDCD1* and rs17718883 in *CD274* were associated with PD-L1 immunohistochemical expression in TIICs, whereas rs4143815 in *CD274* appeared to be linked to PD-L1 immunohistochemical expression in ccRCC tumor cells. Furthermore, rs17718883 in the *CD274* gene showed a significant association with OS in patients with ccRCC. Given the limited data currently available in the literature regarding the associations between *PDCD1* and *CD274* gene polymorphisms and ccRCC, we believe that our study contributes to expanding the understanding of the genetic background of this malignancy. As polymorphisms in genes encoding immune checkpoint molecules have not yet been extensively investigated in the context of ccRCC, further studies are needed to validate the associations observed in our research.

## Figures and Tables

**Table 1:** Allelic and genotypic frequencies of selected single nucleotide polymorphisms (SNPs) in clear cell renal cell carcinoma (ccRCC) cases.

Gene	Polymorphism	Genotype/Allele	N	%
*PDCD1*	rs11568821	CC	185	77.7%
		CT	18	7.6%
		TT	35	14.7%
		C	388	81.5%
		T	88	18.5%
	rs7603052	CC	31	13.0%
		CT	100	42.0%
		TT	107	45.0%
		C	162	34.0%
		T	314	66.0%
*CD274*	rs4143815	CC	28	11.8%
		CG	93	39.1%
		GG	117	49.2%
		C	149	31.3%
		G	327	68.7%
	rs17718883	CC	105	44.1%
		CT	96	40.3%
		TT	37	15.5%
		C	306	64.3%
		T	170	35.7%

**Table 2:** *PDCD1* and *CD274* polymorphisms and PD-L1 immunohistochemical status in tumor-infiltrating immune cells (TIICs).

Gene	Polymorphism	Genotypes	PD-L1 Positive [n (%)]	PD-L1 Negative [n (%)]	*p*-Value
*PDCD1*	rs11568821	CC	91 (38.24%)	94 (39.50%)	0.730
		CT + TT	24 (10.08%)	29 (12.18%)	
	rs7603052	TT	43 (18.07%)	64 (26.89%)	0.033
		TC + CC	72 (30.25%)	59 (24.79%)	
*CD274*	rs4143815	GG	49 (20.59%)	68 (28.57%)	0.068
		GC + CC	66 (27.73%)	55 (23.11%)	
	rs17718883	CC	59 (24.79%)	46 (19.33%)	0.043

**Table 3:** *PDCD1* and *CD274* polymorphisms and PD-L1 immunohistochemical status in clear cell renal cell carcinoma (ccRCC) tumor cells.

Gene	Polymorphism	Genotypes	PD-L1 Positive [n (%)]	PD-L1 Negative [n (%)]	*p*-Value
*PDCD1*	rs11568821	CC	47 (19.75%)	138 (57.98%)	0.806
		CT + TT	15 (6.30%)	38 (15.97%)	
	rs7603052	TT	27 (11.34%)	80 (33.61%)	0.912
		TC + CC	35 (14.71%)	96 (40.34%)	
*CD274*	rs4143815	GG	23 (9.66%)	94 (39.50%)	0.039
		GC + CC	39 (16.39%)	82 (34.45%)	
	rs17718883	CC	11.34% (n = 27)	32.77% (n = 78)	1.000
		CT + TT	14.71% (n = 35)	41.18% (n = 98)	

**Table 4:** Survival status and *PDCD1* and *CD274* polymorphisms.

Gene	Polymorphism	Genotype	Alive [n (%)]	Dead [n (%)]	*p*-Value
*PDCD1*	rs11568821	CC	107 (59.12%)	74 (40.88%)	0.099
		CT	6 (33.33%)	12 (66.67%)	
		TT	19 (54.29%)	16 (45.71%)	
	rs7603052	CC	17 (54.84%)	14 (45.16%)	0.504
		CT	58 (59.79%)	39 (40.21%)	
		TT	58 (54.21%)	49 (45.79%)	
*CD274*	rs4143815	CC	15 (53.57%)	13 (46.43%)	0.693
		CG	50 (53.76%)	43 (46.24%)	
		GG	69 (59.48%)	47 (40.52%)	
	rs17718883	CC	67 (65.04%)	36 (36.96%)	<0.001
		CT	46 (47.91%)	50 (52.09%)	
		TT	20 (54.05%)	17 (45.95%)	

**Table 5:** Demographic and clinical characteristics of clear cell renal cell carcinoma cases.

Characteristics	Clear Cell Renal Cell Carcinoma Cases
Number of tumor samples [n (%)]	238 (100.0%)
Age, years [mean ± SD]	63.9 ± 10.0
Sex [n (%)]	
Female	83 (34.9%)
Male	155 (65.1%)
Type of operation [n (%)]	
Radical nephrectomy	146 (61.3%)
Partial nephrectomy (NSS)	92 (38.7%)
Tumor location [n (%)]	
Right kidney	132 (55.5%)
Left kidney	106 (44.5%)
Tumor size, cm (mean ± SD)	5.4 ± 3.1
Tumor stage [n (%)]	
pT1	145 (60.9%)
pT2	20 (8.4%)
pT3	72 (30.3%)
pT4	1 (0.4%)
WHO/ISUP grading [n (%)]	
G1	94 (39.5%)
G2	83 (34.9%)
G3	27 (11.3%)
G4	34 (14.3%)

## Data Availability

The original contributions presented in this study are included in the article and Supplementary Materials. Further inquiries can be directed to the corresponding author.

## Supplementary Materials

The following supporting information can be downloaded at: https://www.mdpi.com/article/10.3390/ijms27083435/s1.

## Abbreviations

The following abbreviations are used in this manuscript:

PD-1	programmed death protein 1
PD-L1	programmed cell death ligand 1
RCC	renal cell carcinoma
ccRCC	clear cell renal cell carcinoma
SNP	single nucleotide polymorphism
TIICs	tumor-infiltrating immune cells
OS	overall survival
3′-UTR	3′-untranslated region
miRNA	microRNA
FDR	false discovery rate
SD	standard deviation
ICI	immune checkpoint inhibitor
