# Supplementary material for: Association Between Polymorphisms in Genes Encoding PD-1/PD-L1 Molecules and Clinicopathological Features in Clear Cell Renal Cell Carcinoma
Source: Int J Mol Sci. 2026 Apr 11;27(8):3435. doi: 10.3390/ijms27083435 (PMC13116213; doi:10.3390/ijms27083435)

Supplemental Figure S1. Genotypic frequencies of selected single nucleotide polymorphisms (SNPs) in clear cell renal cell carcinoma cases.

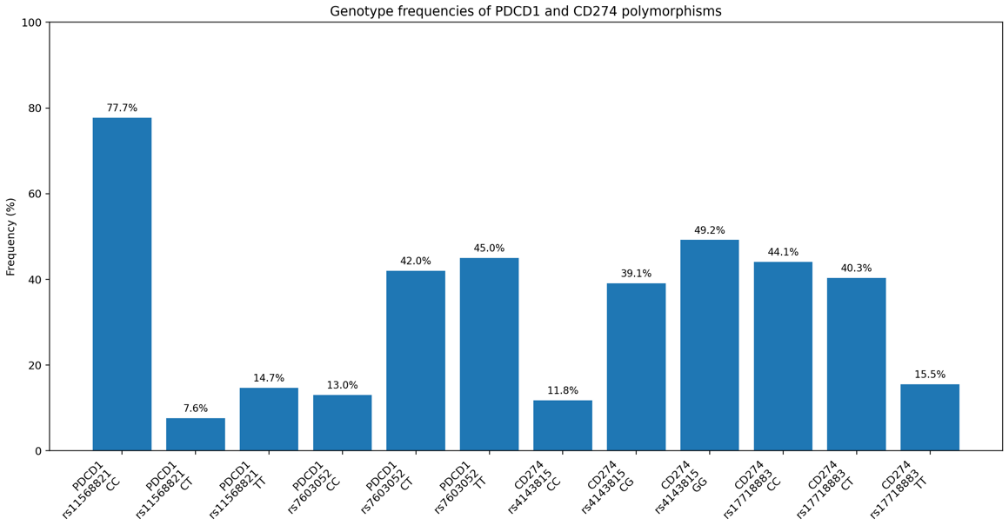

Supplemental Figure S2. Allelic frequencies of selected single nucleotide polymorphisms (SNPs) in clear cell renal cell carcinoma cases.

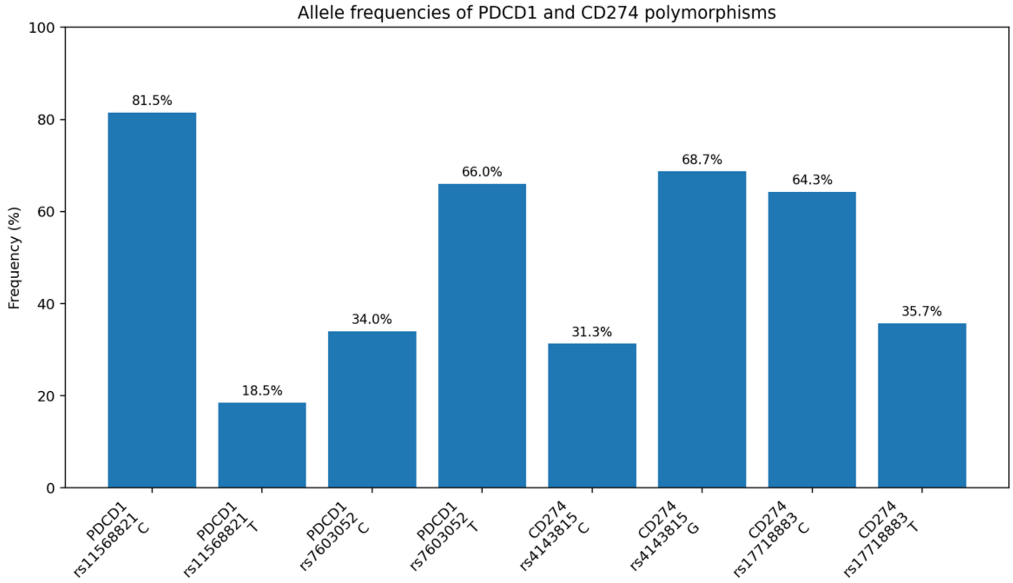

Supplemental Figure S3. PDCD1 and CD274 polymorphisms and PD-L1 immunohistochemical status in tumor-infiltrating immune cells (TIICs).

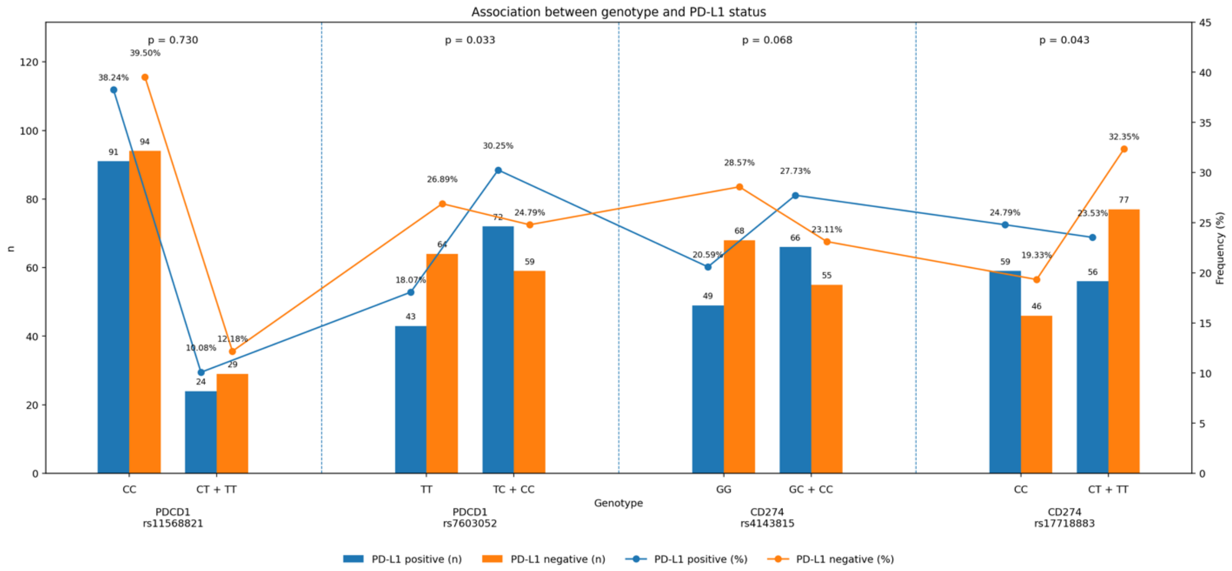

Supplemental Figure S4. PDCD1 and CD274 polymorphisms and PD-L1 immunohistochemical status in clear cell renal cell carcinoma tumor cells.

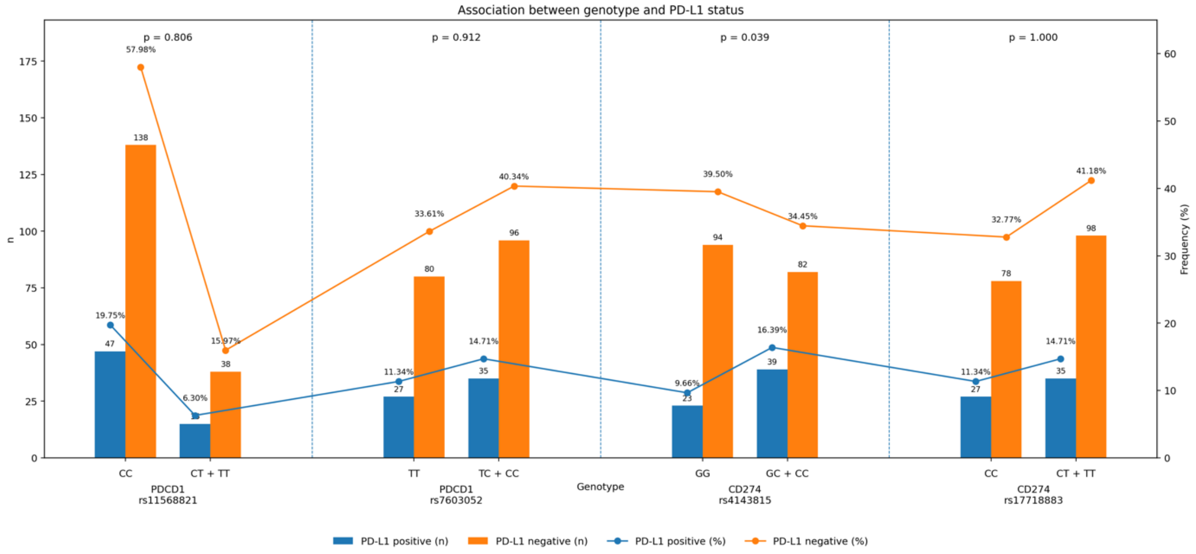

Supplemental Figure S5. Survival status and PDCD1 and CD274 polymorphisms.

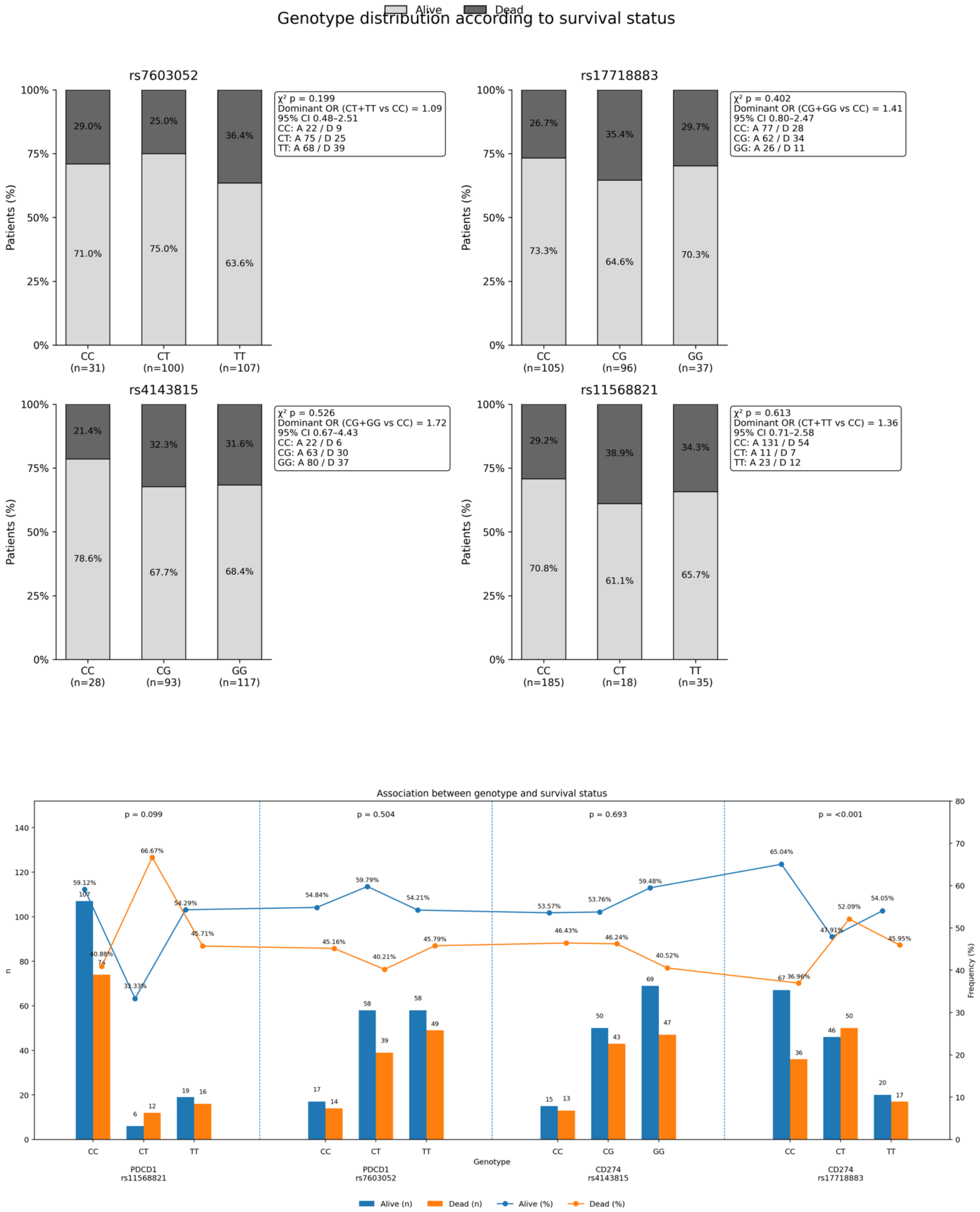

Supplement: Supplementary file 1 [file ijms-27-03435-s001.zip › Supplemental Figures.pdf]
