# Supplementary material for: Association Between Polymorphisms in Genes Encoding PD-1/PD-L1 Molecules and Clinicopathological Features in Clear Cell Renal Cell Carcinoma
Source: Int J Mol Sci. 2026 Apr 11;27(8):3435. doi: 10.3390/ijms27083435 (PMC13116213; doi:10.3390/ijms27083435)
Supplement: Supplementary file 1 [file ijms-27-03435-s001.zip › Supplemental Tables.pdf]

Supplemental Table S1. PDCD1 and CD274 polymorphisms and patients' gender.

Gene	Polymorphism	Genotypes	Female [n (%)]	Male [n (%)]	p-value
PDCD1	rs11568821	CC	63 (26.47%)	122 (51.26%)	0.7396
		CT + TT	20 (8.40%)	33 (13.87%)	
	rs7603052	TT	38 (15.97%)	69 (28.99%)	0.9597
		TC+CC	45 (18.91%)	83 (36.13%)	
CD274	rs4143815	GG	42 (17.65%)	75 (31.51%)	0.8495
		GC+CC	41 (17.23%)	80 (33.61%)	
	rs17718883	CC	32 (13.45%)	73 (30.67%)	0.8495
		CT+TT	51 (21.43%)	82 (34.45%)	

Supplemental Table S2. PDCD1 and CD274 polymorphisms and patients' age.

Gene	Polymorphism	Genotypes	≤60 yo [n (%)]	61–70 yo [n (%)]	>70 yo [n (%)]	p-value
PDCD1	rs11568821	CC	61 (25.63%)	80 (33.61%)	44 (18.49%)	0.2096
		CT + TT	14 (5.88%)	20 (8.40%)	19 (7.98%)	
	rs7603052	TT	29 (12.18%)	50 (21.01%)	28 (11.76%)	0.3273
		TC+CC	46 (19.33%)	50 (21.01%)	35 (14.71%)	
CD274	rs4143815	GG	36 (15.13%)	51 (21.43%)	30 (12.61%)	0.8888
		GC+CC	39 (16.39%)	49 (20.59%)	33 (13.87%)	
	rs17718883	CC	33 (13.87%)	46 (19.33%)	26 (10.92%)	0.8389
		CT+TT	42 (17.65%)	54 (22.69%)	37 (15.55%)	

Supplemental Table S3. PDCD1 and CD274 polymorphisms and tumor size.

Gene	Polymorphism	Genotypes	< 4 cm [n (%)]	4–7 cm [n (%)]	7–10 cm [n (%)]	> 10 cm [n (%)]	p-value
PDCD1	rs11568821	CC	67 (28.15%)	61 (25.63%)	47 (19.75%)	10 (4.2%)	0.739
		CT + TT	18 (7.56%)	18 (7.56%)	12 (5.04%)	5 (2.1%)	
	rs7603052	TT	37 (15.55%)	39 (16.39%)	25 (10.5%)	6 (2.52%)	0.801
		TC+CC	48 (20.17%)	40 (16.81%)	34 (14.29%)	9 (3.78%)	
CD274	rs4143815	GG	43 (18.07%)	41 (17.23%)	25 (10.5%)	8 (3.36%)	0.683
		GC+CC	42 (17.65%)	38 (15.97%)	34 (14.29%)	7 (2.94%)	
	rs17718883	CC	38 (15.97%)	35 (14.71%)	27 (11.34%)	5 (2.1%)	0.853
		CT+TT	47 (19.75%)	44 (18.49%)	32 (13.45%)	10 (4.2%)	

Supplemental Table S4. PDCD1 and CD274 polymorphisms and presence of angioinvasion.

Gene	Polymorphism	Genotypes	Angioinvasion present [n (%)]	Angioinvasion absent [n (%)]	p-value
PDCD1	rs11568821	CC	29 (12.18%)	156 (65.55%)	0.0592
		CT + TT	15 (6.30%)	38 (15.97%)	
	rs7603052	TT	20 (8.40%)	87 (36.55%)	1.0000
		TC+CC	24 (10.08%)	107 (44.96%)	
CD274	rs4143815	GG	19 (7.98%)	98 (41.18%)	0.4768
		GC+CC	25 (10.50%)	96 (40.34%)	
	rs17718883	CC	23 (9.66%)	82 (34.45%)	0.2990
		CT+TT	21 (8.82%)	112 (47.06%)	

Supplemental Table S5. PDCD1 and CD274 polymorphisms and WHO/ISUP grade.

Gene	Polymorphism	Genotypes	G1 + G2 [n (%)]	G3 + G4 [n (%)]	p-value
PDCD1	rs11568821	CC	138 (74.6%)	47 (25.4%)	0.8820
		CT + TT	39 (73.6%)	14 (26.4%)	
	rs7603052	TT	83 (77.6%)	24 (22.4%)	0.3068
		TC+CC	94 (71.8%)	37 (28.2%)	
CD274	rs4143815	GG	87 (74.4%)	30 (25.6%)	0.9970
		GC+CC	90 (74.4%)	31 (25.6%)	
	rs17718883	CC	77 (73.3%)	28 (26.7%)	0.9168
		CT+TT	100 (75.2%)	33 (24.8%)	

Supplemental Table S6. PDCD1 and CD274 polymorphisms and pathologic stage (pT) of the primary tumor.

Gene	Polymorphism	Genotypes	pT1 + pT2 [n (%)]	pT3 + pT4 [n (%)]	p-value
PDCD1	rs11568821	CC	134 (72.4%)	51 (27.6%)	0.0523
		CT + TT	31 (58.5%)	22 (41.5%)	
	rs7603052	TT	70 (65.4%)	37 (34.6%)	0.2375
		TC+CC	95 (72.5%)	36 (27.5%)	
CD274	rs4143815	GG	85 (72.6%)	32 (27.4%)	0.2745
		GC+CC	80 (66.1%)	41 (33.9%)	
	rs17718883	CC	68 (64.8%)	37 (35.2%)	0.1747
		CT+TT	97 (72.9%)	36 (27.1%)	
